# The prevalence and associated factors of non-communicable disease risk factors among civil servants in Ibadan, Nigeria

**DOI:** 10.1371/journal.pone.0203587

**Published:** 2018-09-13

**Authors:** Abisola T. Olawuyi, Ikeola A. Adeoye

**Affiliations:** Department of Epidemiology and Medical Statistics, Faculty of Public Health, College of Medicine, University of Ibadan, Ibadan, Nigeria; University of Maiduguri College of Medical Sciences, NIGERIA

## Abstract

**Background:**

Non-communicable diseases (NCDs) have become a global public health problem, which threatens Sub-Sahara Africa (SSA) including Nigeria. Civil servants are at risk of NCDs because of the stressful and sedentary nature of their work. The study aimed to determine the prevalence and associated factors of the major risk factors of NCDs among civil servants in Ibadan, Nigeria.

**Methods:**

A cross-sectional study was conducted among 606 civil servants in Oyo State using a two-stage cluster sampling technique. The WHO Stepwise approach was used to assess the behavioural and metabolic risk factors. Anthropometric (weight, height, waist and hip circumferences), blood pressure and biochemical measurements (fasting blood sugar) were obtained. Prevalence rates and 95% confidence intervals were calculated. Multivariate logistic models with adjusted odds ratios and their 95% confidence intervals were used to assess the associated factors of NCD risk factors. Multiple Poisson regression was also performed to determine the effects of certain socio-demographic factors on the clustering of NCD risk factors.

**Results:**

The mean age of the civil servants was 43.0±10.3 and 53.8% were males. The prevalence estimates and 95% confidence intervals of the risk factors were 6.5% (95% CI:4.5–8.5) for current smoking, 7.8% (95% CI:5.1–10.5) for harmful use of alcohol, 62.2% (95% CI:58.2–66.2) for low physical activity, 69.7% (95% CI:66.0–73.4) for insufficient fruit and vegetable intake, 37.1% (95% CI:33.2–41.0) for abdominal obesity, 57.3% (95% CI:53.3–61.3) for overweight and obesity, 33.1% (95% CI:29.3–36.8) for raised blood pressure and 7.1% (95% CI:5.0–9.1) for raised blood sugar. Over 75% of the population had at least two NCD risk factors and the study participants had an average of 3 NCD risk factors 3.01 (95% CI: 2.88–3.14) The female gender was significantly associated with an increased risk for abdominal obesity (AOR 27.9; 95% CI: 12.09–64.6) and being overweight or obese (AOR 6.78; 95% CI: 3.53–13.01), but was protective of smoking (AOR 0.21; 95% CI: 0.07–0.61) and binge drinking (AOR 0.04; 95% CI: 0.01–0.45). Also, the risk of hypertension increased with age– 30–39 years (AOR 12.29; 95% CI: 1.06–141.8), 40–49 years (AOR 14.28; 95% CI: 1.10–181.4) and 50 years and above (AOR 32.43; 95% CI: 2.44–413.7). Raised blood pressure was a strong correlate for having raised blood sugar (AOR 5.63; 95% CI: 1.48–21.3). Increasing age (IRR 1.02; 95% CI: 1.01–1.02) and being female (IRR 1.36; 95% CI: 1.23–1.49) were also important predictors of the clustering of risk factors.

**Conclusion:**

The feminization (i.e. the preponderance of risk factors among the females) and clustering of non-communicable disease risk factors were observed among Oyo State civil servants. Our findings highlight the high prevalence of cardio-metabolic risk factors among the working class. Hence the need for targeted preventive and therapeutic interventions among this population.

## Introduction

Non-communicable diseases (NCDs) have become the leading cause of morbidity and mortality world-wide. In 2012, out of 56 million deaths globally, NCDs accounted for 38 million and 28 million of these NCD deaths occurred in low and medium income countries [1]. These deaths have been projected to increase from 38 million in 2012 to 52 million by 2030 [1], particularly in low and middle income countries, which already bear an appreciable burden of communicable diseases (a double burden of disease). For example, in 2012, NCD mortality for the African region alone was 28 million [1]. Most African countries are undergoing an epidemiological transition, which is a shift from a pattern of predominantly infectious diseases to that of chronic, non-communicable diseases. This is as a result of urbanization, industrialization, increased life expectancy and the adoption of western lifestyle characterized by reduced physical activity and dietary changes from foods rich in fruits and vegetables to refined, energy-dense and fatty foods.

Current evidence shows that four major groups of diseases namely cardiovascular diseases, cancers, respiratory diseases and diabetes mellitus account for 82% of all NCD deaths, [1]. These diseases share four common behavioural risk factors (tobacco use, excess alcohol consumption, unhealthy diet and physical inactivity) and four metabolic risk factors (elevated blood pressure, overweight and obesity, hyperglycaemia and hyperlipidaemia). The World Health Organization (WHO) has recommended the surveillance of NCDs and their risk factors to inform the implementation of appropriate public health strategies. The WHO Stepwise approach is a standardized methodology for the surveillance of non-communicable diseases (STEPS) [2, 3], although, studies using this approach have just begun to emerge in Nigeria [4–7].

Now it is crucial to pay close attention to NCDs among the working population, because these health challenges can perhaps lead to economic losses, household poverty and reduction in productivity [8]. Potentially, the workplace provides a platform for preventive interventions. This study provides information on the magnitude of the NCD risk factors among civil servants in Ibadan and a baseline for monitoring the trends, guiding decision making, and implementing appropriate interventions.

## Materials and methods

### Study design and sampling technique

A cross-sectional survey was conducted among 606 civil servants in Oyo State Secretariat, Ibadan, using a two stage cluster sampling technique. Ibadan is the capital city of Oyo State and the third largest city in Nigeria, after Lagos and Kano (National Population Commission, 2009). It covers an area of 3,080 km^2^ with coordinates 7°23΄47″N and 3°55′0″E. Ibadan consists of 11 Local Government Areas (LGAs).

The Oyo State Secretariat is the administrative headquarters of the State Government located at Agodi, Ibadan North Local Government Area, with coordinates 7°24΄35″N and 3°54′28″E. The Secretariat accommodates the Governor’s office, State House of Assembly, government agencies and 20 state ministries. The civil service lies at the center of public administrative structure. The study population were the state civil servants in Ibadan. The number of civil servants in each ministry ranged from 150 to 250 from which an average of 50 civil servants were selected from each of the 12 ministries randomly selected to participate in the study. In each ministry, 1–2 study participants were chosen consecutively from the offices until the sample size was reached. Non-consenting individuals, contract staff, pregnant women and those absent at the time of the study probably due to ill health.

The sample size was calculated using the sample size determination for prevalence study, based on a precision of 5%, a Z statistics for the level of confidence of 95% and prevalence of overweight of 27% [5]. The minimum sample size was 316 and was adjusted to 592 to account for non-response rate of 20% and design effect.

### Data collection

#### Step 1

The study was conducted using the WHO Stepwise approach for non-communicable diseases, which consists of three steps. Step 1 involves the use of semi structured, pretested, interviewer-administered questionnaires adapted from the WHO NCD risk factor surveillance questionnaire [3] ] to collect information on socio-demographic characteristics (age, gender, marital status, level of education, income level and job cadre), behavioral risk factors (current smoking, harmful use of alcohol, low intake of fruits and vegetables, and low physical activity) and metabolic risk factors (overweight and obesity, abdominal obesity, elevated blood pressure and fasting blood sugar). *The details of the measures of behavioural and biological risk factors can be found in the attached supplementary file* (“S1 File”).

**Job cadre**: This was based on the salary scale of civil servants, with those between grades 1–6 classified as junior staff, 7–12 as mid-level staff, 13–17 as senior staff.

**Tobacco use**: This was defined as current use of any tobacco product in either smoked or smokeless form within 30days prior to study.

**Harmful use of Alcohol (Binge drinking)**: This pertained to any male who reported having ≥5 drinks or to any female having ≥ 4 drinks on one or more occasion, within 30days prior to study. A drink is defined as a bottle or one glass of wine or a shot of any of the spirit, e.g. gin, red wine.

**Unhealthy diet**: This was described as the low intake of fruits and vegetables that is less than 5 servings per week.

**Insufficient physical exercise**: The physical activity level was assessed using the International Physical Activity Questionnaire (IPAQ) [9]. The International Physical Activity Questionnaire (IPAQ) was used in this study to substitute the Global Physical Activity Questionnaire (GPAQ) because IPAQ has better utility in the Nigerian population than the GPAQ. Besides multiple studies have reported on the reliability and validity of the IPAQ in the Nigerian population [10–12]. Metabolic equivalent (MET) is the ratio of a person’s working metabolic rate relative to the resting metabolic rate. The level of physical activity was assessed using the frequency and the rigor of physical activity per week to arrive at MET-minutes per week. Insufficient physical activity was considered as less than 600 MET-minutes per week. Moderate physical activity was 600–3000 MET-minutes per week and vigorous physical activity was greater than 3000 MET-minutes per week. We also estimated the proportion of study participants that met the WHO recommendation for physical activity: ≥ 75mins or vigorous or 150 mins moderate physical activity per week.

#### Step 2—Anthropometric measurements

Height was measured with a calibrated meter rule and with the participant standing against the wall and facing straight ahead. Weight was measured using an analogue weighing scale (HANA) placed on a flat hard surface. The research assistant ensured the pointer of the machine was on zero. After the study participants had been correctly positioned and the pointer on measuring device became stable, measurement was approximated to the nearest 1kg. Body mass index (BMI) was derived by dividing weight (kg) by height squared (m^2^) and categorized based on the WHO classification: Underweight ≤ 18.5 kg/m^2^, Normal (healthy weight) = 18.5–24.9 kg/m^2^, Overweight = 25.0–29.9 kg/m^2^, Obesity ≥ 30 kg/m^2^ [13]. The waist circumference was determined using a non-extendable measuring tape, which was wound around the subject, midpoint between the lower margin of the last rib and the superior border of the iliac crest. The mid axillary line was used as a reference point. Abdominal obesity was defined as waist circumference (WC) value greater than 102 cm for men and 88 cm for women [14].

#### Step 3 –Biological measures

Blood pressure measurements were done using the *Omron digital automatic blood pressure machine (intelli sense M3W)*. Measurements were taken after respondents had been sitting for a minimum of 10 minutes and three different readings were taken each at three minutes interval. An average of the last two readings was used for analysis. Raised blood pressure was described as systolic BP ≥140 mmHg or / and diastolic BP ≥90 mmHg, including respondents who reported that they had been on antihypertensive treatment [15].

Fasting blood sugar screening was also conducted using the *Accu-check Active* glucometer. Capillary blood was obtained by pricking the respondents on their thumbs. Respondents were informed prior to the day of data collection to fast overnight. Raised blood sugar was defined as a fasting blood sugar ≥ 7.0 mmol/L (126g/dl) or random blood sugar ≥ 11.1 mmol/L (200mg/dl) or being on diabetic medication [16].

### Data processing and analysis

Data were inputted and cleaned using SPSS version 17 and Stata version 12 SE was used for analyses (Data and analytical script are in “S2 and S3 Files”). The dependent variables were abdominal obesity, overweight and obesity, raised blood pressure and raised blood sugar. The independent variables were socio-demographic characteristics and behavioural risk factors. The general characteristics of the study population by socio-demographic and physical parameters are presented in “Table 1”. The categorical variables are presented by absolute numbers and percentages, and means and standard deviation (SD) are used to summarize numeric variables.

**Table 1:** Characteristics of study participants in Ibadan.

	N	n	Frequency
***Socio-demographic factors***			
**Age group**	606		
20–29		67	11.1
30–39		140	23.1
40–49		191	31.5
50+		176	29.0
Missing		32	5.3
**Gender**	606		
Male		326	53.8
Female		280	46.2
**Marital Status**	606		
Single		72	12.3
Currently Married		493	84.4
Previously Married		19	3.3
Missing		22	3.6
**Level of Education**	606		
≤ Primary		33	5.5
Secondary		107	17.7
Post-secondary		439	72.8
Missing		27	4.5
**Income (Naira ₦)**	606		
≤20,999		102	16.8
21,000–60,999		290	47.9
61,000–100,000		98	16.2
>100,000		56	9.2
Missing		60	9.9
**Job Cadre**	606		
Junior		192	31.7
Mid-Level		278	45.9
**Senior**		102	16.8
**Missing**		34	5.6
Physical activity per week*	606		
Adequate		294	48,2
Inadequate		314**	51.8
**Mean and standard deviation of physical characteristics of study participants.**			
Weight(kg)	606	71.1	±12.8
Height (m)	606	1.63	±0.09
Waist (cm)	606	89.9	±12.0
SBP	606	124.9	±19.8
DBP FBS	606 477***	79.3 86.2	±18.2 ± 30.1

* Recommended WHO Physical activity - ≥ 75mins or vigorous or 150 mins moderate physical activity per week

** includes those with missing data

*** others were assessed using random blood sugar

The prevalence of behavioural and biological risk factors are displayed in “Tables 2 and 3”. The overall prevalence estimates and 95% confidence intervals (CI) of behavioural and metabolic risk factors were determined and were also stratified according to age, gender, marital status, level of education, income and job cadre. Chi square for trend was performed and their p-values presented.

**Table 2:** Prevalence (%) and 95% CI of behavioural risk factors for non-communicable diseases among civil servants in Ibadan.

Characteristics	Current Smoking (N)	Harmful use of alcohol (N)	Physical inactivity (N)	Insufficient fruit and vegetable (N)
**Overall prevalence**	6.5 (4.5–8.5)	7.8 (5.1.-10.5)	62.2 (58.2–66.2)	69.7 (66.0–73.4)
**Age group**				
20–29	9.1 (2.1–16.1)	9.1 (0.5–17.7)	49.2 (37.0–61.5)	75.0 (64.3–85.7)
30–39	9.2 (4.2–14.1)	7.6 (2.1–13.1)	58.4 (49.9–67.0)	74.6 (67.2–82.0)
40–49	6.1 (2.6–9.7)	5.2 (1.1–9.3)	65.7 (58.7–72.8)	72.1 (65.7–78.5)
50+	4.2 (1.1–7.3)	11.0 (5.3–16.7)	67.8 (60.8–75.0)	61.4 (54.1–68.7)
**P for trend**	0.069	0.566	0.005	0.0100
**Gender**				
Male	9.2 (5.9–12.4)	12.6 (8.2–17.0)	63.0 (57.6–68.4)	70.7 (65.6–75.7)
Female	3.3 (1.2–5.6)	1.2 (0.4–3.0)	61.3 (55.3–67.3)	68.6 (63.0–74.1)
**P for trend**	0.005	<0.001	0.686	0.589
**Marital Status**				
Single	8.6 (1.9–15.1)	10.6 (1.7–19.6)	44.9 (33.0–56.8)	75.7 (65.6–85.9)
Currently Married	6.1 (3.9–8.2)	7.3 (4.5–10.3)	63.5 (59.0–67.9)	69.2 (65.0–73.4)
Previously married	10.5 (3.7–24.7)	8.3 (8.1–24.7)	80.0 (59.0–101.0)	63.1 (40.8–85.5)
**P for trend**	0.768	0.529	0.001	0.201
**Level of Education**				
≤ Primary	17.2 (3.2–31.3)	18.2(1.6–34.7)	71.8 (56.0–87.7)	64.5 (47.4–81.7)
Secondary	10.8 (4.7–16.8)	7.7 (1.7–13.8)	62.9 (53.2–72.6)	68.6 (59.6–77.5)
Post-secondary	4.8 (2.8–6.9)	6.8 (3.7–9.8)	60.7 (60.0–65.5)	70.0 (65.7–74.4)
**P for trend**	0.001	0.113	0.244	0.513
**Income**				
< 20,999	13.4 (6.5–20.2)	9.5 (2.7–16.2)	57.0 (46.8–67.1)	78.8 (70.7–86.9)
21,000–60,999	6.2 (3.3–9.1)	5.2 (2.0–8.3)	63.3 (57.6–69.1)	68.1 (62.6–73.6)
61,000–100,000	2.2 (0.8–5.2)	11.5 (2.7–20.3)	61.0 (51.8–71.6)	69.1 (59.8–78.3)
>100,000	5.7 (0.6–12.0)	7.6 (2.8–18.2)	66.0 (52.7–79.3)	55.3 (42.2–68.5)
**P for trend**	0.016	0.833	0.377	0.007
**Job Cadre**				
Junior	12.0 (7.3–16.8)	9.7 (4.7–14.8)	60.2 (53.0–67.4)	74.7 (68.5–81)
Middle level	3.4 (1.2–5.7)	5.7 (2.3–9.2)	65.1 (59.3–71.0)	67.5 (61.9–73.1)
Senior level	3.1 (0.3–6.6)	11.8 (2.8–20.7)	61.9 (52.1–71.6)	65.0 (55.5–74.4)
**P for trend**	<0.001	0.933	0.627	0.061

**Table 3:** Prevalence (%) and 95% CI of biological risk factors for non-communicable diseases among civil servants in Ibadan.

Characteristics	Abdominal obesity	Raised blood pressure	Overweight or obesity	Raised blood sugar
**Overall** **Prevalence**	37.1 (33.2–40.1)	33.1 (29.3–36.8)	57.3 (53.3–61.3)	7.1 (5.0–9.1)
**Age group**				
20–29	21.2 (11.3–31.2)	9.1 (2.1–16.1)	38.5 (26.5–50.4)	3.0 (-1.1–7.1)
30–39	25.4 (18.1–32.7)	18.8 (12.3–25.4)	47.4 (39.0–55.9)	5.7 (1.8–9.6)
40–49	43.4 (36.3–50.5)	34.7 (27.9–41.5)	68.4 (61.8–75.1)	4.7 (1.7–7.7)
50+	42.6 (35.3–50.0)	49.7 (42.3–57.1)	59.2 (51.9–66.5)	13.1 (8.0–18.1)
**p for trend**	0.001	<0.001	0.001	0.011
**Gender**				
Male	10.2 (6.9–13.5)	31.0 (25.9–36.0)	42.4 (37.0–47.8)	7.4 (4.5–10.2)
Female	68.3 (62.8–73.8)	35.6 (30.0–41.3)	74.8 (69.7–80.0)	6.8 (3.8–9.7)
**p for trend**	0.001	0.219	0.001	0.976
**Marital Status**				
Single	21.1 (11.5–30.7)	12.9 (4.9–20.8)	35.7 (24.4–47.0)	2.8 (- 0.8–19.0)
Currently Married	37.0 (32,7–41.3)	35.1 (30.9–39.3)	59.8 (55.4–64.2)	7.5 (5.2–9.8)
Previously married	63.2 (40.8–85.5)	52.6 (29.5–75.7)	57.9 (35.0–80.7)	5.3 (-5.1–15.6)
**p for trend**	0.001	0.001	0.001	0.182
**Level of Education**				
Primary	15.1 (2.7–27.6)	59.3 (43.6–77.5)	34.3 (17.6–51.1)	9.1 (-0.8–19.0)
Secondary	43.9 (34.5–53.4)	37.4 (30.9–39.3)	55.2 (45.7–64.8)	9.3 (3.8–14.9)
Post-secondary	36.4 (31.8–41.0)	29.9 (25.6–34.2)	58.3 (53.7–63.0)	6.1 (3.9–8.4)
**p for trend**	0.340	0.003	0.022	0.715
**Income**				
< 20,999	27.5 (18.7–36.1)	28.4 (19.6–37.2)	42.6 (32.9–52.2)	6.8 (1.9–11.8)
21,000–60,999	39.8 (34.1–45.5)	31.3 (26.0–36.7)	56.3 (50.5–62.1)	6.2 (3.4–9.0)
61,000–100,000	26.8 (17.9–35.7)	42.9 (31.9–51.7)	63.9 (54.3–73.6)	7.1 (2.0–12.3)
>100,000	44.6 (31.5–57.8)	35.7 (23.0–48.4)	69.6 (57.5–81.8)	12.5 (3.7–21.3)
**p for trend**	0.306	0.078	0.001	0.018
**Job Cadre**				
Junior	30.0 (23.5–36.5)	25.3 (19.1–31.5)	48.4 (41.2–55.6)	6.2 (2.8–9.7)
Middle level	42.4 (36.5–48.2)	31.3 (32.1–43.6)	60.4 (54.6–66.2)	6.1 (3.3–8.9)
Senior level	32.6 (23.5–41.9)	35.3 (25.9–44.6)	67.3 (58.1–76.5)	11.8 (5.5–18.1)
**p for trend**	0.290	0.095	0.110	0.174

Multivariate logistic regression analyses (Adjusted odds ratios and 95% CI) were performed to identify the associated factors of overweight and obesity, abdominal obesity, raised blood pressure and raised blood sugar and to adjust for confounders like the socio-demographic variables and behavioural risk factors in “Tables 4 and 5”. Multiple Poisson regression analyses (Incidence rate ratios and 95% CI) were performed to determine the effects of certain co-variates on the clustering of NCD risk factors in “Table 6”.

**Table 4:** Predictors of behavioural risk factors of non-communicable diseases among Oyo State civil servants.

Variables	Smoking	Binge drinking	Low fruit & vegetable intake	Physical inactivity
**Age group**				
20–29	1	1	1	1
30–39	1.22 (0.24–6.35)	0.99 (0.12–9.10)	1.73 (0.67–4.45)	0.90 (0.37–2.17)
40–49	1.82 (0.29–11.3)	1–19 (0.09–16.18)	1.44 (0.53–3.93)	1.13 (0.44–2.95)
50+	0.53 (0.63–4.42)	2.35 (0.16–35.19)	0.99 (0.35–2.81)	1.15 (0.43–3.13)
**Gender**				
Male	1			
Female	0.21(0.07–0.61)*	0.04 (0.01–0.45)*	1.08 (0.70–1.67)	0.88 (0.57–1.34)
**Marital status**				
Single	1	1	1	1
Currently Married	0.73 (0.16–3.23)	0.34 (0.04–2.80)	1.05 (0.44–2.52)	2.14 (0.94–4.83)
Previously married	4.41 (0.39–50.12)	4.4 (0.14–138.3)	0.98 (0.24–4.04)	3.54 (0.72–17.47)
**Level of education**				
≤ Primary	1	1	1	1
Secondary	0.40 (0.08–1.95)	0.49 (0.09–2.66)	1.08 (0.38–3.07)	0.80 (0.43–1.60)
Post-secondary	0.20 (0.04–1.27)	0.57 (0.08–3.85)	0.96 (0.34–2.75)	0.82 (0.28–2.44)
**Income**				
< 20,999	1	1	1	1
21,000–60,999	0.55 (0.21–1.45)	0.85 (0.24–3.05)	0.59 (0.29–1.18)	0.83 (0.43–1.60)
61,000–100,000	0.20 (0.22–1.77)	2.17 (0.29–16.1)	0.62 (0.24–1.60)	0.71 (0.28–1.77)
>100,000	0.26 (0.02–4.42)	0.63 (0.03–10.56)	0.36 (0.12–1.06)	1.04 (0.35–3.10)
**Job cadre**				
Junior	1	1	1	1
Middle level	0.40 (0.14–1.15)	0.62 (0.17–2.35)	0.85 (0.49–1.48)	1.03(0.62–1.75)
Senior level	0.64 (0.58–6.93)	0.45 (0.50–4.11)	1.06 (0.81–9.59)	0.95(0.39–2.52)

*Statistically significantly at p<0.05

**Table 5:** Predictors of behavioural risk factors of non-communicable diseases among Oyo State civil servants.

Variables	Overweight & obesity	Abdominal obesity	Raised blood pressure	Raised blood sugar
**Age group**				
20–29	1	1	1	1
30–39	1.09 (0.27–4.33)	0.82 (0.14–4.95)	12.29(1.06–141.8)*	0.51 (0.04–6.9)
40–49	1.05 (0.28–4.72)	1.42(0.21–9.45)*	14.28(1.10–184.4)*	0.29 (0.12–5.3)
50+	0.47 (0.10–2.24)	1.18(0.17–8.08)*	32.43 (2.44–413.7)*	0. 55 (0.3–10.06)
**Gender**				
Male	1	1	1	1
Female	6.78 (3.53–13.01)*	27.9 (12.06–64.60)*	0.90 (0.40–2.02)	1.10 (0.22–5.48)
**Marital status**				
Single	1	1	1	1
Currently Married	0.73 (0.19–2.85)	2.24 (0.38–13.04)*	0.65 (0.12–3.55)	1.41 (0.09–21.1)
Previously married	0.31 (0.27–3.45)	3.74 (0.19–72.14)	1.44 (0.10–21.06)	-
**Level of education**				
≤ Primary	1	1	1	1
Secondary	0.73 (0.21–2.67)	2.86 (0.35–23.10)*	0.21 (0.05–0.87)*	3.58 (0.26–49.0)
Post-secondary	0.59 (0.16–2.24)	3.09 (0.37–26.02)*	0.14 (0.03–0.63)*	2.25 (0.14–36.0)
**Income**				
< 20,999	1	1	1	1
21,000–60,999	1.35 (0.54–3.11)	1.67 (0.53–5.22)*	0.58 (0.22–1.51)	0.30 (0.07–1.34)
61,000–100,000	1.94(0.52–7.27)	1.06 (0.20–5.75)*	0.96 (0.25–3.72)	0.02 (0.01–0.94)*
>100,000	7.90(1.52–41.09)*	8.02 (1.18–54.47)*	0.75 (0.15–3.60)	0.16 (0.01–3.58)
**Job cadre**				
Junior	1	1	1	1
Middle level	1.70(0.83–3.46)	0.99 (0.40–2.42)	1.46 (0.66–3.27)	0.68 (015–3.03)
Senior level	1.38(0.36–5.31)	0.66 (0.13–3.41)	0.90 (0.22–3.54)	5.04 (0.27–95.7)
Current smoking	1.42(0.53–3.83)	0.40 (0.78–2.42)	0.64 (0.19–2.17)	0.34 (0.03–4.12)
Binge drinking	1.38(0.49–3.68)	0.28 (0.25–3.10)	2.98 (1.00–8.91)	2.52 (0.37–17.19)
Low fruit and vegetables	0.75(0.42–1.37)	0.57 (0.26–1.24)	0.91 (0.48–1.72)	1.51 (0.43–5.27)
Low physical activity	3.82(1.03–3.21)*	1.88 (0.90–3.92)*	0.78 (0.42–1.44)	2.40(0.70–8.21)
Overweight & obesity	-	-	1.90 (0.89–4.04)	4.57(0.86–25.70)**
Abdominal obesity	-	-	2.05 (0.82–5.08)	1.09(0.20–6.07)
Raised blood pressure	-	-	-	5.63 (1.48–21.3)*

*Statistically significantly at p<0.05.

**Table 6:** Mean number of risk factors for NCDs and the effect of socio-demographic factors on the clustering of risk factors.

Covariates	Mean number of risk factors (95%CI)	IRR [95% CI]	Robust standard errors
**Overall**	3.01 (2.88–3.14)	-	-
**Age in years**		1.02 (1.01–1.02)*	0.003
20–29	2.13 (1.80–2.46)		
30–39	2.58 (2.33–2.82)		
40–49	3.20 (2.98–2.43)		
50+	3.45 (3.20–3.70)		
**Gender**			
Male	2.63 (2.46–2.80)	1	0.065
Female	3.46 (3.28–3.65)	1.36 (1.23–1.49)*	
**Marital Status**			
Single	2.18 (1.82–2.53)	1	
Currently Married	3.09 (2.94–3.65)	1.16 (0.94–1.42)	0.122
Previously married	3.78 (2.81–4.34)	1.05 (0.78–1.41)	0.156
**Level of Education**			
< Primary	3.15 (2.62–3.68)	1	
Secondary	3.23 (2.91–3.55)	0.96 (0.77–1.20)	0.107
Post-secondary	2.92 (2.77–3.07)	0.98 (0.79–1.23)	0.110
**Income**			
< 20,999	2.75 (2.47–3.04)	1	
21,000–60,999	2.99 (2.80–3.43)	0.95 (0.84–1.08)	0.063
61,000–100,000	3.11 (2.79–3.43)	1.01 (0.85–1.19)	0.086
>100,000	3.25 (2.81–3.68)	1.01 (0.83–1.23)	0.100

*Statistically significantly at p<0.05.

### Ethical consideration

Ethical approval for the study was obtained from the Oyo State Ministry of Health Ethical Committee. Permission to conduct the study was obtained from the Permanent Secretary of each Ministry. Informed written consent was obtained from individual respondents before commencement of the research. The study protocol and conduct adhered to the principles laid down in the Declaration of Helsinki. All participants were assessed and informed on their individual behavioural and cardio-metabolic risk factors with a view to assisting them in taking informed decisions on their health. Participants with raised blood pressure and raised blood sugar were informed and advised to seek medical help.

## Results

### General characteristics

The socio-demographic and physical characteristics of the study participants are described in “Table 1”. The mean age was 43.0 ± 10.3 years with more than 60% being 40 years and above. Also, 53.8% were males, and the majority were currently married (84.4%) and had tertiary level education (72.5%). The mean and standard deviations of physical parameters were as follows: weight (71.1 ± 12.8 kg), height (1.63 ± 0.09 m), waist circumference (89.9 ± 12.0 cm), systolic blood pressure (124.9 ± 19.8 mmHg), and diastolic blood pressure (79.3 ± 18.2 mmHg).

### Prevalence of behavioural risk factors

#### Tobacco consumption

The overall prevalence of current smoking was 6.5% (95% CI: 4.5–8.5). The prevalence of smoking among younger civil servants aged less than 40 (9.1%, 95% CI: 4.2–14.1) was higher than among older civil servants aged 50 and above 4.2% (95% CI: 1.1–7.3). The prevalence of smoking was also significantly higher (p = 0.005) among males 9.2% (95% CI: 5.9–12.4) than females 3.3% (95% CI: 1.2–5.6). The prevalence of smoking had an inverse relationship with the levels of education, income and job cadre the highest occurrence being among those with lowest level of education 17.2% (95% CI: 3.2–31.2), lowest income earners (13.4%, 95% CI: 6.5–20.2) and the lowest cadre of staff (12.1%, 95% CI: 7.3–16.8).

#### Alcohol consumption

The general prevalence of alcohol consumption was 28.6% (95% CI: 24.8–32.4) among respondents while the occurrence of binge drinking was 7.8% (95% CI: 5.1–10.5). A significantly higher prevalence was also observed among men 12.6% (95% CI: 8.2–17.0) compared to the level among women 1.2% (95% CI: 0.4–3.0).

#### Physical inactivity

Low physical activity was prevalent 62.2% (95% CI: 58.2–66.2) among the study participants. Low levels of physical activity significantly increased with age: 49.2% (95% CI: 37.0–61.5) for 20–29 years, 58.4% (95% CI: 49.9–67.0) for 30–39 years, 65.7% (95% CI: 58.7–72.8) for 40–49 years, 67.8% (95% CI: 60.8–75.0) for 50 and above. Also, low physical activity was more common among those currently married 63.5% (95% CI: 59.0–67.9) or previously married 80.0% (95% CI: 59.0–101.0) compared with the single 44.9% (95% CI: 33.0–56.8). Low physical activity was commonest among individuals with the highest income 66.0% (95%: CI 52.1–79.3) compared with the low income group 57.0% (95%: CI 46.8–67.1).

#### Fruit and vegetable intake

The low intake of fruits and vegetables was high among the respondents 69.7% (95% CI: 69.7–73.4). Whilst the prevalence was high across all age groups, it was lowest among those aged 50 and above 61.4% (95% CI: 54.1–68.7). Low intake of fruits and vegetables significantly decreased by the level of income– 78.8% (95% CI: 70.7–86.9) for <₦20,999; 68.1% (95% CI: 62.6–73.6) for <₦21,000 - ₦60,999; 69.1% (95% CI: 59.8–78.3) for <₦61,000 – ₦100,000; and 55.3% (95% CI: 42.2–68.5) for >₦100,000.

### Prevalence of biological risk factors

#### Raised blood pressure

The mean systolic blood pressure was 124.9mmHg ± 19.8, while the mean diastolic blood pressure was 79.3 ± 18.2. The prevalence of raised blood pressure was 33.1% (95% CI: 29.3–36.8) and the prevalence significantly increased with age– 9.1% (95% CI: 2.1–16.1) for 20–29; 18.8% (95% CI: 12.3–25.4) for 30–39; 34.7% (95% CI: 27.9–41.5) for 40–49; 49.7% (95% CI: 42.3–57.1) for respondents aged 50 and above. We also observed an inverse relationship between raised blood pressure and the level of education of respondents, with a higher prevalence among those with primary education 59.3% (95% CI: 43.6–77.5) compared to those with post-secondary education 29.9% (95% CI: 25.6–34.2).

#### Abdominal obesity

Overall prevalence of abdominal obesity was 37.1% (95% CI: 33.2–40.1) and the mean waist circumference was 89.9cm ± 12.0. The prevalence of abdominal obesity was significantly higher among women at 68.3% (95% CI: 62.8–73.8) than among men at 10.2% (95% CI: 6.9–13.5). The prevalence also significantly increased with age– 21.2% (95% CI: 11.3–31.2) for 20–29; 25.4% (95% CI: 18.1–32.7) for 30–39; 43.4% (95% CI: 36.3–50.5) for 40–49; 42.6% (95% CI: 35.3–50.0) for respondents aged 50 and above.

#### Overweight and obesity

The prevalence of overweight and obesity among civil servants in Ibadan was 31.2% and 26.1% respectively. Being overweight or obese had a positive relationship with age, gender, marital status and income. Three-quarters of females were overweight or obese: 74.8% (95% CI: 69.7–80.0), respondents above 40 years had the highest prevalence, 68.4% (95% CI: 61.7–75.1) and those with the highest income (>₦100,000 monthly) had the highest prevalence rate 69.6% (95% CI: 57.5–81.8).

#### Raised blood sugar

The general prevalence of raised blood sugar in the study population was 7.1% (95% CI: 5.0–9.1). The prevalence among males: 7.4% (95% CI: 4.5–10.2) compares with the prevalence among females: 6.8% (95% CI: 3.8–9.7). The highest prevalence was noted among those aged 50 and above: 13.1% (95% CI: 8.0–18.1), among those with the highest income (>₦100,000 monthly) 12.5% (95% CI: 3.7–21.3), and among those holding senior positions 11.8% (95% CI: 5.5–18.1).

### Associations with non-communicable disease risk factors

The associated factors of behavioural and cardio-metabolic risk factors are represented in Tables 4 and 5. The odds of smoking and binge drinking were lower among females, as they were five times less likely to smoke (AOR: 0.21, 95% CI 0.07–0.61) and 25 times less likely to be involved in binge drinking (AOR:0.04 95% CI 0.003–0.45) than the males. However, being previously married increased the likelihood of smoking (AOR: 4.41, 95% CI 0.39–50.17) and binge drinking (AOR: 4.4, 95% CI 0.14–138.3). Contrastingly, being currently married was protective against smoking (AOR: 0.73, 95% CI 0.16–3.23) and binge drinking (AOR: 0.34, 95% CI 0.04–2.80).

The predictors of overweight and obesity in the study were being female (AOR: 6.78, 95% CI: 3.53–13.01), low physical activity (AOR: 3.82, 95% CI 1.03–3.21) and high income (AOR: 7.90, 95% CI 1. 52–41.09). Generally, the factors associated with abdominal obesity were older age, female gender, being currently married, level of education and low physical activity. Females were almost 30 times more likely than males to have abdominal obesity (AOR: 27.9, 95% CI 12.06–64.6). Likewise, those currently married were twice more likely to have abdominal obesity than singles (AOR: 2.24, 95% CI: 0.38–13.04). The likelihood of abdominal obesity also increased as the level of education increased as those with secondary school education (AOR: 2.86, 95% CI: 0.35–23.10) and post-secondary school education (AOR: 3.09, 95% CI: 0.37–26.02) were three times more likely to have abdominal obesity compared those with only primary school education. Also, low physical activity increased the odds twice (AOR: 1.88, 95% CI: 0.90–3.92).

The associated factors of raised blood pressure were age, binge drinking and overweight/obesity. The likelihood of raised blood pressure increased with age: 30–39 years (AOR: 13.08, 95% CI: 1.10–155.3), 40–49 years (AOR: 13.26, 95 CI: 0.99–177.7), 50 years and above (AOR: 36.95, 95% CI: 2.67–510.6). Those with overweight/obesity were twice more at risk of raised blood pressure (AOR: 2.21, 95% CI: 1.02–4.79). The significant predictors of raised blood sugar were overweight/obesity (AOR: 4.57, 95% CI: 0.83–25.07) and raised blood pressure (AOR: 5.75, 95% CI: 1.51–21.9).

### Clustering of behavioral and biological risk factors in study participants

**Fig 1:**
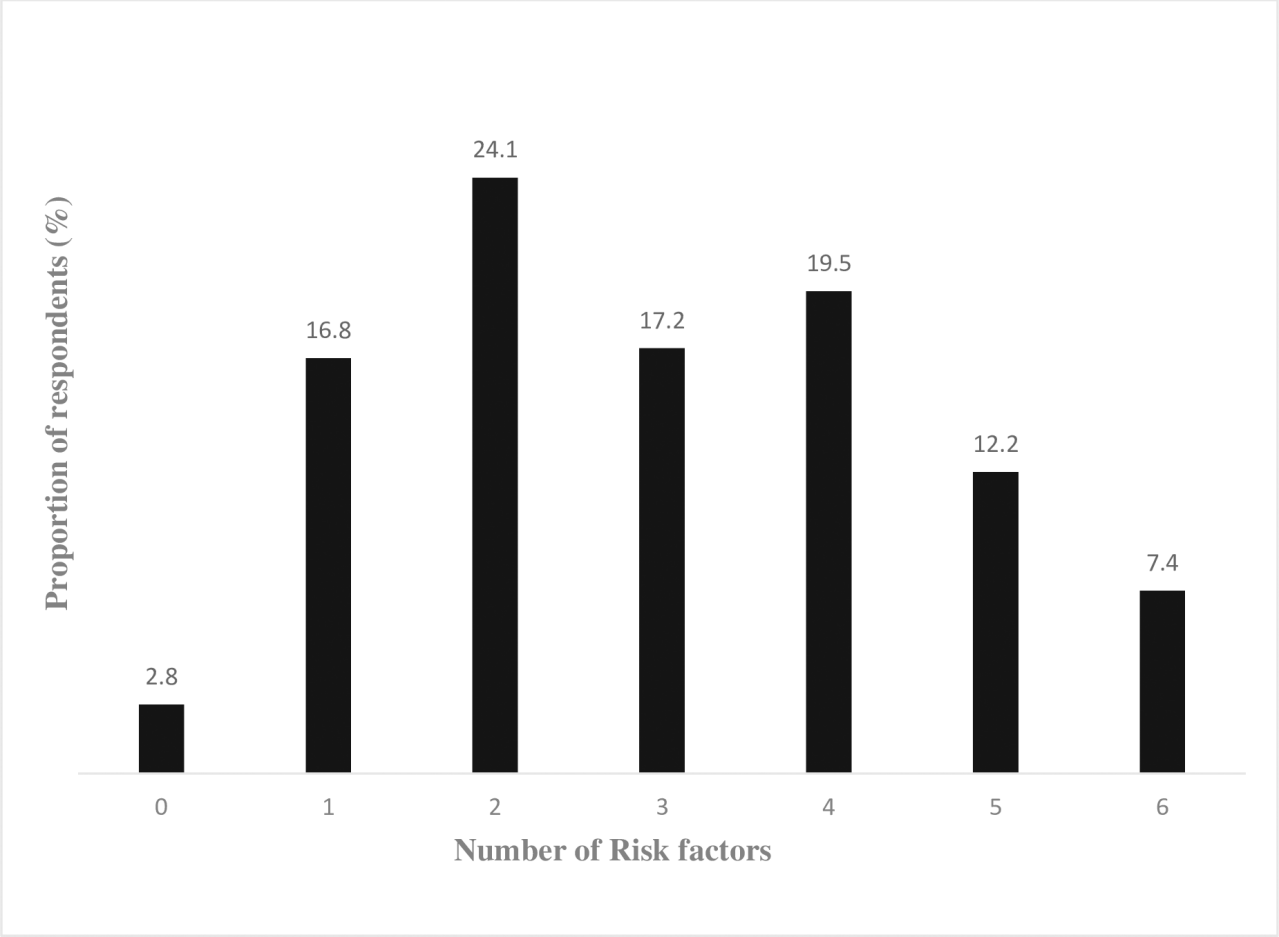
Prevalence of multiple risk factors among civil servants in Ibadan.

The prevalence of multiple NCD risk factors among the civil servants is shown in (Fig 1) and only a negligible proportion of the civil servants (2.8%) were free of NCD risk factors. Over 75% of the population had at least two NCD risk factors and on average the study participants had 3 NCD risk factors (95% CI: 2.88–3.14) Table 6.

Table 6 shows the result of the multiple Poisson regression used to predict the effect of socio-demographic factors on the clustering of risk factors. For every 1 year increase in age there was a 1.02 (95% CI: 1.01–1.02) times increase in the clustering of risk factors. There was also a 36% increase in clustering of risk factors among females 1.36 (95% CI: 1.23–1.49) compared with the males.

## Discussion

Non-communicable diseases, an ‘epidemic in slow motion’, have been projected to be a leading cause of morbidity and mortality in Nigeria by 2030 [17]. The surveillance of NCD risk factors is one of the key strategies advocated to tackle these emerging public health concerns, particularly in low and middle income countries. Most of the studies assessing the prevalence and the predictors of NCDs have come from Asian countries [18–21], whereas such studies have only just begun to emerge in Nigeria [4–7]. Hence our study investigated the prevalence and associated factors of behavioural risk factors (current smoking, harmful alcohol, physical inactivity and unhealthy diet) and biological risk factors (overweight/obesity, abdominal obesity, raised blood pressure and raised blood sugar) among civil servants in Oyo State Nigeria using the WHO Stepwise approach. We found that certain behavioural and biological risk factors were prevalent among our study population, particularly among women. Notably, physical inactivity and low fruit and vegetable intake were the most common risk factors in our study population.

Tobacco use, the leading cause of morbidity and mortality globally that claims about 6 million lives annually [22–23], was the least common risk factor (6.4%) in our study. Similarly, low prevalence rates have been reported among the working class in some other parts of Nigeria [5–7]. Generally, smoking is not a common habit among the Nigerian adult population compared with other African countries–[24–26]. Agaku *et al* [27] in a study assessing poly-tobacco use in 44 countries during 2008–2012, reported that Nigeria had the lowest prevalence of current tobacco use (5.6%) compared with South Africa (20.4%), India (34.4%) and Bangladesh (43.2%). One plausible reason for the low prevalence of smoking among the Nigerian population is the high level of religious involvement that has been shown to influence smoking behaviour. The inverse relationship between high religious involvements and cigarette smoking have been reported by some researchers [28–29]. Nevertheless, we noted in our study that the prevalence was higher among the younger worker and among males. This finding suggests that these groups of persons should be the target for tobacco control interventions, particularly the young people. The Surgeon General Reports (2012) found that the majority of adult smokers initiated the habit of smoking before the age of 18 years [30]. It is little wonder that young people have been the target of the tobacco industry through their advertisement and promotional activities, as well as through their emerging tobacco products, which aim at young people. For example, flavored cigarettes and mentholated smokeless tobacco product such as *ZIP metholated snus* are now available in the Nigerian market [31]. We also noted that the prevalence is much higher among males than among females who were about five times less likely (AOR- 0.21) to indulge in the habit. This pattern has been well documented [24–27]: tobacco use by females is culturally unacceptable in Nigeria just as the harmful use of alcohol, is also culturally unacceptable as noted by other researchers in Nigeria [4, 5, 6]. Alcohol consumption and harmful use of alcohol were reported in more than a quarter of (28.6%) and 7.2% of our study population respectively. We found that being male was a significant predictor of harmful alcohol use. Approximately 2.3 million die each year from the harmful use of alcohol, accounting for about 3.8% of all deaths in the world. More than half of these deaths occur from NCDs including cancers, cardiovascular disease and liver cirrhosis [1].

In our study, physical inactivity was high (62.2%), which agrees with previous studies in Nigeria [5, 6, 32]. We perhaps underestimated the level of physical inactivity in our study because of the subjective method of assessment in the use of self-reported questionnaires–(International Physical Activity Questionnaire—IPAQ). For future studies, there is the need to use more objective means of assessing physical activity like pedometers and accelerometers. Also, we found that the likelihood of physical inactivity increased with age and income. High income affords individuals the means for a lifestyle that unduly relies on motorized transport, labour-saving devices, and indulgence in sedentary pastimes like watching television and video games. Besides, sedentary occupations like civil service jobs also involve prolonged hours of sitting. Therefore, workplace interventions that encourage physical activity at work should be encouraged. For instance, the prohibition of commercial vehicles and motorcycles within the Oyo State Secretariat encourages some level of physical activity in the workplace. This may explain why the level of physical inactivity is much lower than that of States’ civil service, such as that of Kaduna State [5]. Physical inactivity is now a public health concern hence the WHO recommends that adults should engage in at least 150 minutes of moderate-intensity aerobic physical activity or at least 75 minutes of vigorous-intensity aerobic physical activity during the week [33]. We found that less than 50% our study population met this recommendation.

Daily and sufficient intake of fruits and vegetables protects against NCDs. Currently, 1.7 million deaths worldwide are attributable to low intake of fruits and vegetables [34]. Low intake of fruits and vegetables was the commonest risk factor of NCDs in the study (69.7%). This risk factor is also underestimated, because intake of fruits and vegetables in this study was assessed using at least 5 servings per week instead of the recommended minimum 5 servings of fruits and vegetables per day. Aryal and coworkers (2015), in their nationwide survey of the burden of NCD risk factors in Nepal, assessed adequate fruits and vegetables intake as 5 servings per day and found that almost the entire population (99%) had inadequate intake of fruits and vegetables [18]. Although, very few researchers have investigated the intake of fruits and vegetables in Africa, Maimela et al (2016) in the Limpopo Province of South Africa, reported a high prevalence (86%) of insufficient intake of fruits and vegetables [26]. Also, we noted that inadequate intake of fruits and vegetables reduced correspondingly with reduced level of income. Actually, fruits are expensive in the urban areas in Nigeria because fruits and vegetables are sourced from the rural areas, indicating the need to promote programmes that will increase the production of fruits and vegetables to make them more available and affordable as several developed countries (Canada, Japan, Denmark, etc.) have done to implement programmes that promote fruits and vegetables through mass media campaign and school health programmes [35–36]. Additionally, Zeba and co-workers (2012) in Burkina Faso have demonstrated an association between the traditional dietary pattern, which consisted mainly green leafy vegetables and cereals, had lower risk of cardio metabolic risk factors. [37].

Overweight and obesity were found in more than half of the study population and was associated with increasing age and income. The prevalence rate of overweight and obesity among civil servants in Oyo State (57.3%) is similar to the rates reported among civil servants in Kaduna [5] and Abakaliki [32] but is much lower than the rate in Lagos (70.7%) which has higher socio- economic status [38]. The occurrence of overweight and obesity is partly attributable to the sedentary nature of civil service work [physical inactivity (OR = 3.82)], which is usually associated with prolonged hours of sitting, minimal energy expenditure, snacking on energy-dense foods, including sugar-sweetened beverages, and infrequent consumption of homemade foods. Prolonged hours of sitting has been associated with increased risk of raised blood sugar, cardiovascular diseases, and cancers [38–40]. Besides, fast food or restaurant prepared meals are usually calorie-dense with high levels of sugar, salt and oils to enhance the taste and induce people, for profit reasons, to consume portions in excess of their needs. Hence, there is the need to promote the provision of healthy meals and regulate the services of canteens. Importantly, gender was a strong predictor of being overweight and obese with the females having 7 times higher the odds (OR = 6.78) of being overweight and obese than males. Apart from the factors explained, weight gain from previous pregnancies and postpartum weight retention in women who have had children may contribute to obesity in these women. High income (OR = 7.90) was a significant factor of overweight and obesity because it affords an undue reliance on automated devices like washing machines, dish washers, passive transport and sedentary past times [41–42].

Abdominal obesity, which occurred in close to two-fifths of the study population (37.1%) was prevalent in our study population. Actually, abdominal obesity is a more ominous sign for future NCDs, because it is an indicator of visceral obesity, which is associated with higher cardio-metabolic risk [13]. Females were about 30 times more likely than the males (AOR = 27.9) to have abdominal obesity. The other associated issues were physical inactivity, being currently married and the level of education.

Raised blood pressure (HT) the major risk factor for cardiovascular diseases (CVDs), which include coronary heart disease, cerebrovascular disease, peripheral vascular disease etc., has become a global concern. This is because CVDs are the leading cause of death globally with an estimated 17.5 million deaths yearly [43]. Unfortunately, most of these deaths (>75%) occur in low and middle income countries. CVDs are also the leading NCD in Nigeria [17]. In our study, we found that about one-third of the respondents had elevated blood pressure (33.1%). This finding has been reported by other Nigerian researchers [4, 44– 45]. The factors associated with raised blood pressure were increasing age, harmful use of alcohol, being overweight or obese. Increasing age has been shown to be a risk factor for raised blood pressure [5, 46–47]. This results from changes occurring within the cardiovascular system like thickening of the arterial wall. Civil servants that were obese and overweight were two times more likely to have raised blood pressure than those who had normal weight [5, 48]. The relationship between hypertension and obesity can be explained by deposit of fat causing narrowing or blockage of the arteries. Thus, the heart does more work in pushing blood against the thickened arterial wall leading to an increase in arterial blood pressure. Additionally, the adipose tissue also contributes to hypertension by their endocrine and paracrine effects on the endothelial cells by producing substances—cytokines leptin and adiponectin which have detrimental effects on the vasculature [49– 50]. However, our study did not show any gender difference in the occurrence of raised blood pressure. Contrariwise, Akinlua and his colleagues (2015) in a systematic review on the current prevalence and pattern of HT in Nigeria, which estimated a crude prevalence that ranged between 2.1–47.2%, stated that most studies in Nigeria reported higher prevalence among males compared to females [51].

Raised blood sugar was the least common biological risk factor in our study with prevalence of 7.1%. However, this prevalence is slightly higher than rates previously reported by researchers in the country [47, 48, 52]. It is important to note that very few studies have assessed the occurrence of raised blood sugar partly because it was previously uncommon among Nigerians [52]. Also, the invasive nature of the screening test (fasting blood sugar), which requires an overnight fast makes the test more difficult to conduct among a healthy study population. Notwithstanding, it is crucial for researchers to assess the magnitude of raised blood sugar within the Nigerian population in order to tackle this emerging public health concern. The factors associated with raised blood sugar included overweight and obesity (AOR = 4.57) and raised blood pressure (AOR = 5.75). Type 2 raised blood sugar and obesity have been described as twin epidemics [53] because obesity is a major predictor of raised blood sugar in developed countries where over 90% of type 2 raised blood sugar patients are either overweight or obese [54].

Remarkably, while we found that current smoking and harmful alcohol were more among males, metabolic risk factors (blood pressure, abdominal obesity, overweight and obesity) were more among females. Finally, a considerable clustering of NCD risk factors was found among our study population with about four-fifth (80.4%) of our study population having two or more risk factors and an average of three risk factors per participant. The proportion of clustering is much higher than what was reported by Oluyombo and co-workers [55] in some semi-urban communities (47.0%) and by Oladapo and colleagues [44] in some rural communities (12.9%), both in South Western Nigeria. In both studies, clustering was found to increase with age and was more among females, which corroborates our findings in which age (IRR = 1.02) and female gender (IRR = 1.36) were the significant predictors for clustering of NCD risk factors. Even in Kenya, Haregu and co-worker [56] also reported clustering of two or more risk factors among their urban slum dwellers (19.8%). Although not fully investigated in this study, metabolic syndrome refers to a cluster of conditions which include increased blood pressure, high blood sugar, abdominal obesity and abnormal cholesterol or triglyceride levels increasing the risk of NCDs. Clustering of NCD risk factors is a predictor of poor progression of disease and premature death. It is also the reason for advocating for an integrated and comprehensive approach towards the control of NCDs rather than employing the known modality of managing NCDs, which targets individual diseases.

### Strengths and limitations

Our work is an important contribution to the surveillance of NCD risk factors in Nigeria. Even though it is not a nationally representative survey, an assessment of civil servants in one of the largest cities in Nigeria can give a minuscular view of the drivers of NCDs within the larger population until the time when nationally representative surveys, which have begun in a few African countries like Kenya [57], would be conducted in Nigeria. Currently, the National response to NCD is just evolving in Nigeria, [17] where most of the indicators like having an operational national policies for NCDs are yet to be implemented. Although, hyperlipidaemia is one of the four metabolic risk factors of NCDs, it was not assessed in this study because of the financial implications. Finally, some of the assessment tools were limited in the level of precision for instance the level of physical activity was assessed using a subjective rather than objective method.

### Conclusion and implication of the study

In conclusion, our study revealed a high prevalence of NCD risk factors among Nigerian civil servants. Overall, inadequate fruits and vegetable intake as well as physical inactivity were the commonest risk factors. We noted that biological risk factors were more common among females than among males. Hence this phenomenon could be described as the “feminization of biological risk factors”. There was also a significant level of clustering of NCD risk factors affecting above 75% of our study population. The clustering of NCD risk factors within any population is a “bad sign”, because it strongly predicts a huge burden of NCDs and the associated adverse outcomes like premature deaths in the future. Importantly, our study highlights the high prevalence of cardio-metabolic risk factors among the working class and thus suggests the need to provide public health interventions against NCDs, perhaps using the workplace as a platform.

## Supporting information

S1 File Survey Questionnaire on NCD risk factors, Ibadan Nigeria. (PDF)

S2 File Raw data of NCD risk factors, Ibadan Nigeria (dta). (DTA)

S3 File Analytical script of NCD risk factors, Ibadan Nigeria. (PDF)
