# Supplementary material for: The prevalence and associated factors of non-communicable disease risk factors among civil servants in Ibadan, Nigeria
Source: PLoS One. 2018 Sep 13;13(9):e0203587. doi: 10.1371/journal.pone.0203587 (PMC6136760; doi:10.1371/journal.pone.0203587)
Supplement: S1 File — (PDF) [file pone.0203587.s001.pdf]

Questionnaire No: _______

SURVEILLANCE OF NCDS RISK FACTORS AMONG CIVIL SERVANTS IN IBADAN.

Respondent consent form

I am Olawuyi, Abisola. A postgraduate student of Epidemiology and Medical Statistics, Faculty of Public Health, College of Medicine, University of Ibadan. In partial fulfilment of the requirements for the award of the degree of Masters in Public Health, I am carrying out a research on Risk Factors for non-communicable diseases among civil servants in Ibadan.

Your sincere response is encouraged as participation in this study is voluntary, absolute anonymity and confidentiality shall be maintained the information provided will only be used for the research purpose if you have accepted to participate in the study.

Please indicate your interest by acknowledging the verbal consent.

Verbal consent; 1. Yes 2. No

_______________________________ ____________________

Signature/Thumbprint of Participant Interview Date

	**SECTION A: SOCIO DEMOGRAPHIC CHARACTERISTICS**	
1	Sex	Male ☐ Female ☐
2	Age (at last birthday)	
3	Your ethnicity	Hausa ☐ Igbo ☐ Yoruba ☐ Others (specify) __________
4	Year of Birth	
5	Highest level of education	☐ No Formal Education ☐ Primary ☐ Secondary ☐Tertiary
6	Marital status	☐ Single ☐Married ☐Widowed ☐ Divorced
7	Average income in a month	☐< 20,000 ☐ 21,000 – 40,000 ☐ 41,000 – 60,000<br>☐ 61,000- 80,000 ☐ 81,000-100,000 ☐ ≥100,000
8	Ministry	

9	Year of assumption of duty	
10	Cadre/grade/level	☐1-6 ☐ 7-12 ☐ 13-17

	**SECTION B NUTRITION AND DIETARY PATTERN**<br>Please state the types of food **(past 24hrs dietary recall**)				
1	Breakfast				
2	Lunch				
3	Dinner				
	In the past 7 days, how many times did you consume any of the below				
		Never	1-2 times	3-4times	5-7times
1	Vegetable e.g. ewedu, eforiro				
2	Starch e.g. Rice, Eba, Yam, amala				
3	Fried Foods e.g. potato chips, chinchin, fried plantain				
4	Dairy Products e.g. Milk, Cheese, Yogurt.				
5	Cereals e.g. Pap, Custard, Oat, Semovita, Wheat.				
6	Fruits e.g. Orange, Apple.				
7	Legumes e.g. Beans, Moinmoin, Soya.				
8	Carbonated Drinks e.g. Coke, Pepsi, Fanta.				
9	Beverages e.g. Malt, Malta Guinness.				
10	Pastries e.g. Meat pie, Dough nuts, hamburger.				
11	Do you regularly add salt to your already cooked meal	Yes ☐ No ☐			

PLEASE TICK/ FILL AS APPROPRIATE

**SECTION C PHYSICAL ACTIVITY**		
24	During the last 7 days, how many times were you at work	
25	How many hours do you normally sit on a typical day	
26	Which of the following best describes you activity at work	☐ Mainly Sedentary (Mostly Sitting with paper or computer)

		☐Predominantly walking at one level (no heavy lifting)<br>☐ Climbing Stairs, Walking Uphill<br>☐ Lifting Heavy
27	Over the last 7days, how many days do you engage in carrying heavy loads, digging, soccer, jogging	___________ days per week (If Not applicable go to next question)
28	How much time did you usually spend doing this activity on a typical day	____________ hours/day<br>____________ minutes/day
29	Thinking over the last 7 days, how many days do you engage in activities<br>like cleaning, washing cloth/car, sweeping, climbing stairs	_____________days per week (If Not applicable go to next question)
30	How much time did you usually spend doing this activity on a typical day	_________ hours per day<br>_________ minutes per day
31	During the last 7 days, on how many days did you walk for at least 10 minutes at a time?	_________ days per week
32	How much time did you usually spend walking on one of those days?	_____ hours per day<br>_____ minutes per day
33	During the last 7 days how much time did you spend sitting (e.g. at desk/computer, visiting friends, driving, watching TV/Movie)	________ hours per day<br>________ minutes per day
**SECTION D: BEHAVIORAL MEASUREMENT PART 1: TOBACCO USE**		
	Do you currently take any tobacco products	☐ Yes ☐ No if no, move to Qu 41
35	If yes, which tobacco product do you take regularly	☐ Cigarette ☐ Marijuana ☐ Snuff ☐ Others (Specify) …………………………
36	If Cigarette, what is the name of the brand	
37	How frequently do you take this	☐ Daily ☐ Weekly ☐ Monthly

38	How frequently have you smoked in the last 30 days	☐ Less than once a week ☐ 1 – 2 times/week<br>☐ 3 – 6 times/week ☐ Everyday
39	If cigarette, how many sticks do you take per day	
40	At what age did you start smoking	
41	In the past, did you ever smoke	☐ Yes ☐ No (if NO, go to section E)
42	How old were you when you stopped smoking	
**SECTION E ALCOHOL CONSUMPTION**		
43	Do you currently take an alcoholic drink	☐ Yes ☐ No If No, go to qu50)
44	Which alcoholic drink do you frequently take (brand name)	
45	At what age did you start drinking	
46	Have you consumed an alcoholic drink within the past 30 days	Yes ☐ No ☐
47	In the last 30 days, how frequently did you take an alcoholic drink	☐ Less than once a week ☐ 1 – 2 times/week<br>☐ 3 – 6 times/week ☐ Everyday
48	During the past 30 days, how many standard alcoholic drinks bottles/can did you have during one drinking occasion	
49	Have you consumed more than 5 alcoholic drinks at a single sitting in the last 30days	Yes ☐ No ☐
50	Did you drink in the past	Yes ☐ No ☐
51	If yes, at what age did you start drinking	
52	How old were you when you stopped	
**SECTION F HIGH BLOOD PRESSURE**		
53	Have you ever been told by a doctor or other health worker that you have high blood pressure or hypertension?	☐ Yes ☐ No
54	Are you currently receiving any of the following treatments/advice for high blood pressure prescribed by a doctor or other health worker?	
	1. Drugs (anti-hypertensive medication) that you have taken in the past two weeks:	☐ Yes ☐ No

	2. Advice to reduce salt intake	☐ Yes ☐ No
	3. Advice or treatment to lose weight	☐ Yes ☐ No
	4. Advice or treatment to stop smoking	☐ Yes ☐ No
	5. Advice to start or do more exercise	☐ Yes ☐ No
55	Is there any family history of hypertension among your first degree relatives	☐ Yes ☐ No
56	Have you ever been told by a doctor or other health worker that you have raised blood sugar or diabetes	☐ Yes ☐ No
57	Are you currently receiving any of the following treatments/advice for diabetes prescribed by a doctor or other health worker?	
	1. Drugs (medication) that you have taken in the past:	☐ Yes ☐ No
	2. Specially prescribed diet	☐ Yes ☐ No
	3. Advice or treatment to lose weight	☐ Yes ☐ No
	4. Advice or treatment to stop smoking	☐ Yes ☐ No
	5. Advice to start or do more exercise	☐ Yes ☐ No
58	Is there any family history of diabetes among your first degree relatives	☐ Yes ☐ No
**SECTION G SCREENING (Tick as appropriate)**		
59	Female	Self-Breast Examination ☐ Yes ☐ No<br>Mammography ☐ Yes ☐ No<br>Pap Smear ☐ Yes ☐ No
60	Male	Prostate ☐ Yes ☐ No<br>If yes which one________________________
61	Is there any family history of cancer	☐ Yes ☐ No
	**SECTION H; AVAILABILITY OF WORKPLACE HEALTH PROMOTION PROGRAMS** ;	
62	Which of the following is available in your place of work	
	Free/subsidized health screening	☐ Yes ☐ No
	Health Walk, Endurance Trek	☐ Yes ☐ No
	Occupational Health Services (e.g. Staff Health Clinic)	☐ Yes ☐ No
	Availability of Health Insurance Scheme	☐ Yes ☐ No
	Health Talk/Free Health Living Advice	☐ Yes ☐ No
	Smoking and Drinking Cessation Facilities	☐ Yes ☐ No

	Stress management/Counselling Session	☐ Yes ☐ No
	Work Free Days/Sick Leave	☐ Yes ☐ No
**FOR OFFICAL USE ONLY**		
63	Weight	
64	Height	
65	Waist Circumference	
66	Hip Circumference	
67	Blood Pressure Reading 1<br>Systolic<br>Diastolic	
68	Blood Pressure Reading 2<br>Systolic<br>Diastolic	
69	Fasting Blood Sugar	
