# Supplementary material for: The prevalence and associated factors of non-communicable disease risk factors among civil servants in Ibadan, Nigeria
Source: PLoS One. 2018 Sep 13;13(9):e0203587. doi: 10.1371/journal.pone.0203587 (PMC6136760; doi:10.1371/journal.pone.0203587)
Supplement: S3 File — (PDF) [file pone.0203587.s003.pdf]

ANALYTICAL SCRIPT
Prevalence and Determinants of Non Communicable Disease Risk Factors among Civil Servants in Ibadan, Nigeria

PREVALENCE

```
ta bo_currsmoke
proportion bo_currsmoke, over(bagegrp50)
proportion bo_currsmoke, over( A1 )
proportion bo_currsmoke, over( bi_marital )
proportion bo_currsmoke, over( education3cat )
proportion bo_currsmoke, over( incomelevelcat )
proportion bo_currsmoke, over( A9 )
ta bo_binge
*// overall prevalences by socio-demographic characteristics ///**
proportion bo_binge, over(bagegrp2)
proportion bo_binge, over(bagegrp50)
proportion bo_binge , over( A1 )
proportion bo_binge , over( bi_marital )
proportion bo_binge , over( bi_marital )
proportion bo_binge , over( education3cat )
proportion bo_binge , over( incomelevelcat )
proportion bo_binge , over( A9 )
proportion bo_lowfruitveg , over(bagegrp2)
proportion bo_lowfruitveg , over(bagegrp50)

proportion bo_lowfruitveg , over( A1 )
proportion bo_lowfruitveg, over( bi_marital )
proportion bo_lowfruitveg , over( bi_marital )
proportion bo_lowfruitveg , over( education3cat )
proportion bo_lowfruitveg , over( incomelevelcat )
proportion bo_lowfruitveg , over( A9 )
proportion bo_lowPA , over(bagegrp2)
proportion bo_lowPA , over(bagegrp50)

proportion bo_lowPA , over( A1 )
proportion bo_lowPA , over( bi_marital )
proportion bo_lowPA , over( bi_marital )
proportion bo_lowPA , over( education3cat )
proportion bo_lowPA , over( incomelevelcat )
proportion bo_lowPA , over( A9 )
proportion bo_overobese , over(bagegrp2)
proportion bo_overobese , over(bagegrp50)

proportion bo_overobese , over( A1 )
proportion bo_overobese , over( bi_marital )
proportion bo_overobese , over( education3cat )
proportion bo_overobese , over( incomelevelcat )
proportion bo_overobese , over( A9 )
proportion bo_abdobese , over(bagegrp50)
proportion bo_abdobese , over( A1 )
proportion bo_abdobese , over( bi_marital )
```

```
proportion bo_abdobese , over( education3cat )
proportion bo_abdobese , over( incomelevelcat )
proportion bo_abdobese , over( A9 )
proportion HT2 , over(bagegrp50)
proportion HT2 , over( A1 )
proportion HT2 , over( bi_marital )
proportion HT2 , over( bi_marital )
proportion HT2 , over( education3cat )
proportion HT2 , over( incomelevelcat )
proportion HT2 , over( A9 )
proportion DM2 , over(bagegrp50)
proportion DM2 , over( A1 )
proportion DM2 , over( bi_marital )
proportion DM2 , over( education3cat )
proportion DM2 , over( incomelevelcat )
proportion DM2 , over( A9 )
*// overall prevalences ///**
proportion bo_abdobese
proportion bo_overobese
proportion bo_lowfruitveg
proportion bo_currsmoke
proportion bo_binge
proportion bo_lowPA
proportion bo_HT
proportion bo_DM
proportion E43
ed bo_abdobese bo_overobese bo_lowfruitveg bo_currsmoke bo_binge bo_lowPA bi_HBP
bo_HT bo_DM clusterisk
tab1 bo_abdobese bo_overobese bo_lowfruitveg bo_currsmoke bo_binge bo_lowPA bi_HBP
bo_HT bo_DM clusterisk
proportion HT2
proportion DM2
```

P VALUE FOR TREND – STATCALC

```
tab2 bagegrp50 bo_currsmoke
tab2 A1 bo_currsmoke
tab2 bi_marital bo_currsmoke
tab2 education3cat bo_currsmoke
tab2 incomelevelcat bo_currsmoke
tab2 A9 bo_currsmoke
***binge
tab2 bagegrp50 binge
tab2 A1 binge
tab2 bi_marital  binge
tab2 education3cat  binge
tab2 incomelevelcat binge
tab2 A9 binge
****PA
tab2 bagegrp50 bo_lowPA
tab2 A1 bo_lowPA
```

```
tab2 bi_marital  bo_lowPA
tab2 education3cat  bo_lowPA
tab2 incomelevelcat bo_lowPA
tab2 A9 bo_lowPA
*** low veg
tab2 bagegrp50 bo_lowfruitveg
tab2 A1 bo_lowfruitveg
tab2 bi_marital  bo_lowfruitveg
tab2 education3cat  bo_lowfruitveg
tab2 incomelevelcat bo_lowfruitveg
tab2 A9 bo_lowfruitveg
***bo_abdobese
tab2 bagegrp50 bo_abdobese
tab2 A1 bo_abdobese
tab2 bi_marital  bo_abdobese
tab2 education3cat  bo_abdobese
tab2 incomelevelcat bo_abdobese
tab2 A9 bo_abdobese
***hypertension
tab2 bagegrp50 bo_HT
tab2 A1 bo_HT
tab2 bi_marital  bo_HT
tab2 education3cat  bo_HT
tab2 incomelevelcat bo_HT
tab2 A9 bo_HT
***bo_overobese
tab2 bagegrp50 bo_overobese
tab2 A1 bo_overobese
tab2 bi_marital  bo_overobese
tab2 education3cat  bo_overobese
tab2 incomelevelcat bo_overobese
tab2 A9 bo_overobese
****bo_DM
tab2 bagegrp50 bo_DM
tab2 A1 bo_DM
tab2 bi_marital bo_DM
tab2 education3cat  bo_DM
tab2 incomelevelcat bo_DM
tab2 A9 bo_DM
```

LOGISTIC REGRESSION

```
logistic bo_currsmoke bagegrp50 A1  bi_marital  education3cat incomelevelcat  A9
logistic bo_currsmoke i.bagegrp50 i.A1  i.bi_marital  i.education3cat i.incomelevelcat  i.A9
logistic bo_binge i.bagegrp50 i.A1  i.bi_marital  i.education3cat i.incomelevelcat  i.A9
logistic bo_lowPA i.bagegrp50 i.A1  i.bi_marital  i.education3cat i.incomelevelcat  i.A9
logistic bo_lowfruitveg i.bagegrp50 i.A1  i.bi_marital  i.education3cat i.incomelevelcat  i.A9
logistic bo_HT i.bagegrp50 i.A1  i.bi_marital  i.education3cat i.incomelevelcat  i.A9
logistic bo_HT i.bagegrp50 i.A1  i.bi_marital  i.education3cat i.incomelevelcat  i.A9
bo_currsmoke bo_binge bo_lowfruitveg bo_lowPA
```

```
logistic bo_HT i.bagegrp50 i.A1  i.bi_marital  i.education3cat i.incomelevelcat  i.A9
i.bo_currsmoke i.bo_binge i.bo_lowfruitveg i.bo_lowPA
logistic bo_HT i.age40cat i.A1  i.bi_marital  i.education3cat i.incomelevelcat  i.A9
i.bo_currsmoke i.bo_binge i.bo_lowfruitveg i.bo_lowPA
logistic bo_HT i.age40cat i.A1  i.bi_marital  i.education3cat i.incomelevelcat  i.A9
i.bo_currsmoke i.bo_binge i.bo_lowfruitveg i.bo_lowPA bo_overobese
logistic bo_HT bagegrp50 i.A1  i.bi_marital  i.education3cat i.incomelevelcat  i.A9
i.bo_currsmoke i.bo_binge i.bo_lowfruitveg i.bo_lowPA bo_overobese
logistic bo_HT i.bagegrp50 i.A1  i.bi_marital  i.education3cat i.incomelevelcat  i.A9
i.bo_currsmoke i.bo_binge i.bo_lowfruitveg i.bo_lowPA bo_overobese
ta F55
ta F55,nol
logistic HT2 i.bagegrp50 i.A1  i.bi_marital  i.education3cat i.incomelevelcat  i.A9
i.bo_currsmoke i.bo_binge i.bo_lowfruitveg i.bo_lowPA bo_overobese i.F55
logistic HT2 i.bagegrp50 i.A1  i.bi_marital  i.education3cat i.incomelevelcat  i.A9
i.bo_currsmoke i.bo_binge i.bo_lowfruitveg i.bo_lowPA bo_overobese bo_abdobese

*logistic bo_overobese i.bagegrp50 i.A1  i.bi_marital  i.education3cat i.incomelevelcat  i.A9
i.bo_currsmoke i.bo_binge i.bo_lowfruitveg i.bo_lowPA
logistic bo_overobese i.bagegrp50 i.A1  i.bi_marital  i.education3cat i.incomelevelcat  i.A9
logistic bo_overobese i.bagegrp50 i.A1  i.bi_marital  i.education3cat i.incomelevelcat  i.A9
i.bo_currsmoke i.bo_binge i.bo_lowfruitveg i.bo_lowPA
logistic bo_abdobese i.bagegrp50 i.A1  i.bi_marital  i.education3cat i.incomelevelcat  i.A9
logistic bo_abdobese i.bagegrp50 i.A1  i.bi_marital  i.education3cat i.incomelevelcat  i.A9
i.bo_currsmoke i.bo_binge i.bo_lowfruitveg i.bo_lowPA
logistic bo_DM i.bagegrp50 i.A1  i.bi_marital  i.education3cat i.incomelevelcat  i.A9
logistic DM2 i.bagegrp50 i.A1  i.bi_marital  i.education3cat i.incomelevelcat  i.A9
i.bo_currsmoke i.bo_binge i.bo_lowfruitveg i.bo_lowPA bo_overobese bo_abdobese HT2
```

POISSON REGRESSION

```
lm clusterisked A2 i.A1 i.bi_marital i.education3cat i.incomelevelcat i.A9, family(poisson)
link(log) vce(robust)
glm clusterisked A2 i.A1 i.bi_marital i.education3cat i.incomelevelcat i.A9, family(poisson)
link(log) eform vce(robust)
glm clusterisked A2 i.A1 i.bi_marital i.education3cat i.incomelevelcat, family(poisson)
link(log) eform vce(robust)
mean clusterisked, over(A1)
mean clusterisked, over( bi_marital )
mean clusterisked, over( education3cat )
mean clusterisked, over( education3cat )
mean clusterisked, over( incomelevelcat )
```
